# Growth of GaN Thin Films Using Plasma Enhanced Atomic Layer Deposition: Effect of Ammonia-Containing Plasma Power on Residual Oxygen Capture

**DOI:** 10.3390/ijms232416204

**Published:** 2022-12-19

**Authors:** Shicong Jiang, Wan-Yu Wu, Fangbin Ren, Chia-Hsun Hsu, Xiaoying Zhang, Peng Gao, Dong-Sing Wuu, Chien-Jung Huang, Shui-Yang Lien, Wenzhang Zhu

**Affiliations:** 1 Xiamen Key Laboratory of Development and Application for Advanced Semiconductor Coating Technology, School of Opto-Electronic and Communication Engineering, Xiamen University of Technology, Xiamen 361024, China; 2 Department of Materials Science and Engineering, National United University, Miaoli 36063, Taiwan; 3 Fujian Provincial Key Laboratory of Nanomaterials, Fujian Institute of Research on the Structure of Matter, Chinese Academy of Sciences, Fuzhou 350002, China; 4 Department of Applied Materials and Optoelectronic Engineering, National Chi Nan University, Nantou 54561, Taiwan; 5 Department of Applied Physics, National University of Kaohsiung, Kaohsiung University Rd., Kaohsiung 81148, Taiwan; 6 Department of Materials Science and Engineering, Da-Yeh University, Changhua 51591, Taiwan

**Keywords:** gallium nitride, plasma-enhanced atomic layer deposition, oxygen

## Abstract

In recent years, the application of (In, Al, Ga)N materials in photovoltaic devices has attracted much attention. Like InGaN, it is a direct band gap material with high absorption at the band edge, suitable for high efficiency photovoltaic devices. Nonetheless, it is important to deposit high-quality GaN material as a foundation. Plasma-enhanced atomic layer deposition (PEALD) combines the advantages of the ALD process with the use of plasma and is often used to deposit thin films with different needs. However, residual oxygen during growth has always been an unavoidable issue affecting the quality of the resulting film, especially in growing gallium nitride (GaN) films. In this study, the NH_3_-containing plasma was used to capture the oxygen absorbed on the growing surface to improve the quality of GaN films. By diagnosing the plasma, NH_2_, NH, and H radicals controlled by the plasma power has a strong influence not only on the oxygen content in growing GaN films but also on the growth rate, crystallinity, and surface roughness. The NH and NH_2_ radicals contribute to the growth of GaN films while the H radicals selectively dissociate Ga-OH bonds on the film surface and etch the grown films. At high plasma power, the GaN film with the lowest Ga-O bond ratio has a saturated growth rate, a better crystallinity, a rougher surface, and a lower bandgap. In addition, the deposition mechanism of GaN thin films prepared with a trimethylgallium metal source and NH_3_/Ar plasma PEALD involving oxygen participation or not is also discussed in the study.

## 1. Introduction

Gallium nitride (GaN) has attracted much attention as a third-generation wide bandgap semiconductive material for high-performance devices, such as light emitting diodes (LEDs) [1,2,3] and high electron mobility transistors (HEMTs) [4,5] due to its wide bandgap and high breakdown electric field [6,7]. A good transparency and chemical stability also make GaN a candidate material for the carrier transport layer in solar cells. Furthermore, for applications in solar cells, InGaN/GaN heterojunctions with very excellent photovoltaic properties can also be fabricated by forming In_x_Ga_1-x_N on GaN layers [8,9]. Therefore, the preparation technology of high-quality GaN thin films has been widely studied. Metal-organic chemical vapor deposition (MOCVD) [10,11], molecular beam epitaxy (MBE) [12,13], pulsed laser deposition (PLD) [14,15], and atomic layer deposition (ALD) [16,17,18,19] are most commonly used to prepare GaN films. Although GaN films with high electron mobility have been obtained with MOCVD and MBE epitaxy, the extremely high deposition temperature exceeds the tolerance temperature of fluorine-doped tin oxide (FTO) transparent electrodes, which limits the application of GaN in solar cells [20]. Among these deposition techniques, ALD technique can precisely control the film thickness at the atomic level and grow large-area, uniform, conformal, pinhole-free, and stoichiometric thin films at low temperatures. Plasma-assisted ALD technology, also known as plasma-enhanced atomic layer deposition (PEALD), further improves the quality of GaN films by providing highly reactive radical species [21].

Many studies have reported the growth and the applications of GaN thin films prepared using PEALD at low temperatures [20,22,23,24,25,26,27]. However, oxygen incorporation into the PEALD–GaN films has been commonly observed in many studies and leads to degradation of the film quality. The source of oxygen is attributed to the unavoidable presence of residues or moisture in the process chamber during deposition [28,29,30]. Additionally, since Ga easily reacts with oxygen to form gallium oxide, this makes the control of oxygen an important issue for the preparation of GaN films by PEALD. Not only for GaN films, research on reducing the oxygen incorporation into nitride films during PEALD process is limited. Saurabh Karwal et al., reported PEALD-HfN_x_ films prepared using CpHf (NMe_2_)_3_ precursor and H_2_ plasma, where an external radio frequency (RF) substrate bias was applied to have a high ion impact energy, thereby reducing the Hf-O bonds [31]. However, the oxygen-influenced growth mechanism and oxygen elimination during the growth of GaN film process by PEALD with a NH_3_ containing plasma has not been fully investigated. Moreover, the effect of oxygen content on the properties of GaN films has not been completely studied also.

In this study, GaN thin films were deposited using PEALD with a NH_3_ containing plasma. The optical emission spectroscopy (OES) was used to investigate the NH_3_ radicals of the plasma generated during the deposition process. The effect of oxygen capture by controlling the plasma power is discussed. In addition, the effects of oxygen elimination on the properties and optical properties of GaN thin films under different NH_3_-containing plasma powers is also discussed. A GaN growth mechanism involving oxygen has therefore been proposed.

## 2. Results and Discussion

**Figure 1:**
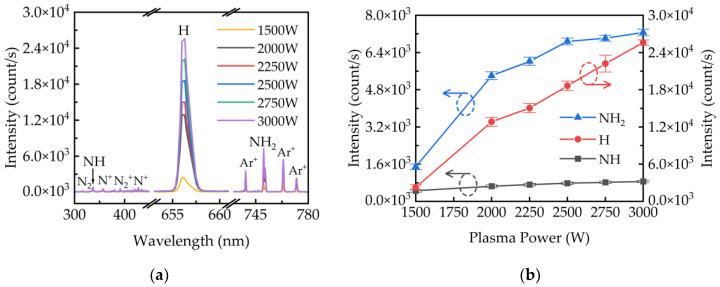
(**a**) The plasma emission spectra obtained at various plasma powers. (**b**) The plot of emission intensity of H, NH, and NH_2_ radicals as a function of plasma powers.

Figure 1a shows the OES results of plasma diagnosis during the PEALD deposition of GaN. The emission spectra obtained at various plasma powers from 1500 to 3000 W show signals of Ar^+^, NH, NH_2_, H, N^+^, $N_{2}^{+}$, and N_2_ radical species. The H radical was observed to have the highest emission intensity among other radical species. In addition, there is no emission of any O-related radical species during the growth of GaN films. The emission intensity of all radical species increases with increasing the plasma powers. For H-containing radicals, Figure 1b plots the increasing emission intensity of H, NH, and NH_2_ radicals as a function of plasma power. It is known that the dissociation of H-NH_2_ and H-NH requires an energy of 435 kJ/mol and 377 kJ/mol, respectively [32]. The formation of NH radical (812 kJ/mol) requires more energy than NH_2_ radical (435 kJ/mol) resulting in a much lower emission intensity of NH than NH_2_. A low power at 1500 W has a low dissociation of the NH_3_/Ar plasma and leads to the lowest emission intensity. At powers higher than 1500 W, a significant increase in the emission intensity of NH_2_ and H was observed, which indicates that NH_3_ is efficiently dissociated in the plasma with increasing power. Furthermore, the H emission increases linearly and has the most pronounced increase compared to NH and NH_2_ emissions. The increase in NH_2_ emission intensity drops slightly between 2000 and 2500 W, and a further drop was observed at 2500 to 3000 W. The results indicate that the dissociation of NH_2_ becomes saturated at higher plasma powers and that H radicals dominate the growth of GaN films.

**Figure 2:**
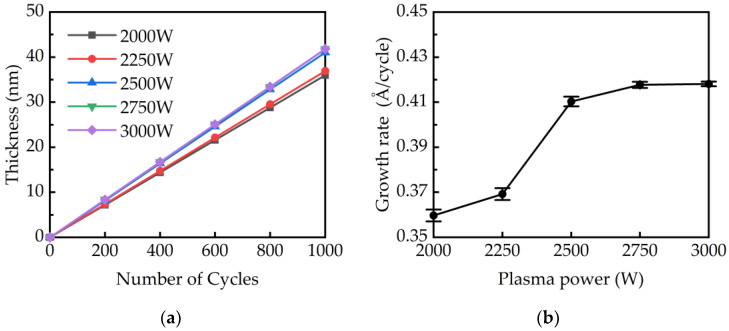
(**a**) The GaN thickness obtained after cycles at different plasma powers. (**b**) The growth rate of GaN calculated at different plasma powers.

As shown in Figure 2a, the thickness of the GaN films linearly increased with the number of growth cycles. The growth rates of GaN films at different plasma powers were calculated and plotted in Figure 2b. The increase in growth rate is due to the increase in radical concentration at higher plasma powers. At 1500 W, the GaN thickness is 15.56 nm after 1000 cycles while the growth rate is 0.16 Å/cycle. The growth rate obtained at 1500 W were excluded because the plasma dissociation rate and plasma density at 1500 W were too low to grow GaN films. At low plasma power of 2000 to 2250 W, the slopes in Figure 2a are closed with an average growth rate at 0.365 Å/cycle. Between powers of 2250 and 2500 W, the growth rate rises sharply to 0.41 Å/cycle. For a power higher than 2500 W, the growth rate reaches a saturation value at 0.42 Å/cycle and has a close slope in Figure 2a. The results indicate that the growth rate was affected by the dissociated radicals in the plasma. A low plasma power lower than 2250 W leads a low dissociation rate and results a low NH_2_ intensity and a low growth rate. As the power increases from 2250 to 2500 W, the NH_2_ radicals are significantly enhanced as discussed in Figure 1b and increase the growth rate as shown in Figure 2b. For the power higher than 2500 W, as shown in Figure 1b, a slow increase in NH_2_ and a significant increase in H radicals have the effect of reducing growth and increase etching, respectively, leading to a saturated growth rate of GaN films.

For the deposition mechanism of GaN thin films prepared by PEALD using TMGa metal source and NH_3_/Ar plasma, a series of chemical reactions in NH_3_/Ar plasma and on sample surface are proposed.

In NH_3_/Ar plasma:

Ar + NH_3_ + (e^−^)_Plasma_ → Ar^+^ + NH + NH_2_ + H + N^+^ + N_2_^+^ + N_2_ + e^−^, (1)

On sample surface:

(NH + NH_2_ + H)_Plasma_ + Ŝ-H → Ŝ-NH_2_ + H_2 (g)_, (2)

1st Half Reaction:

Ŝ-(NH_2_)_x_ + Ga(CH_3_)_3 (g)_ → Ŝ-(N-Ga(CH_3_)_3-x_)_x_ + CH_4 (g)_ (x = 1, 2), (3)

2nd Half Reaction:

Ŝ-(N-Ga(CH_3_)_3-x_)_x_ + H → Ŝ-(N-GaH_3-x_)_x_ + CH_4 (g)_, (4)

Ŝ-(N-GaH_3-x_)_x_ + (NH + NH_2_ + H)_plasma_ → Ŝ-(N-Ga(NH_2_)_3-x_)_x_ + H_2 (g)_, (5)

where Ŝ and g represent the substrate and the gas phase, respectively, while the plus sign represents the ionized state of the species.

Before deposition, the native oxides on the surface of the samples were removed with 2% HF solution, and then the surface was passivated with hydrogen [33,34]. In the NH_3_/Ar plasma, Equation (1) describes that the NH_3_/Ar gas mixture is excited to produce a plasma containing Ar^+^, NH, NH_2_, H, N^+^, N_2_^+^, and N_2_ radical species, which is confirmed by the OES measurement. As shown in Equation (2), the H-passivated sample surface (Ŝ-H) reacts with the NH and NH_2_ radicals in the plasma to form Ŝ-NH_2_ groups on the surface. It is noted that the Ŝ-NH_2_ groups acts as the reaction sites of TMGa during the following ALD process. The growth of GaN in the ALD process is described by two half-reactions, as shown in Equations (3)–(5). In the 1st half-reaction, as shown in Equation (3), TMGa adsorbs on the surface and bonds with Ŝ-NH_2_ groups to produce Ŝ-(N-Ga(CH_3_)_3-x_)_x_, where x represents the number of reaction sites of TMGa (x = 1, 2). In the following 2nd half-reaction, Ŝ-(N-Ga(CH_3_)_3-x_)_x_ reacts with H radicals in the plasma to form an intermediate species of Ŝ-(N-GaH_3-x_)_x_ [35]. Subsequently, NH and NH_2_ radicals replace the H in Ŝ-(N-GaH_3-x_)_x_ to form Ŝ-(N-Ga(NH_2_)_3-x_)_x_ leading to the growth of GaN monolayer.

**Figure 3:**
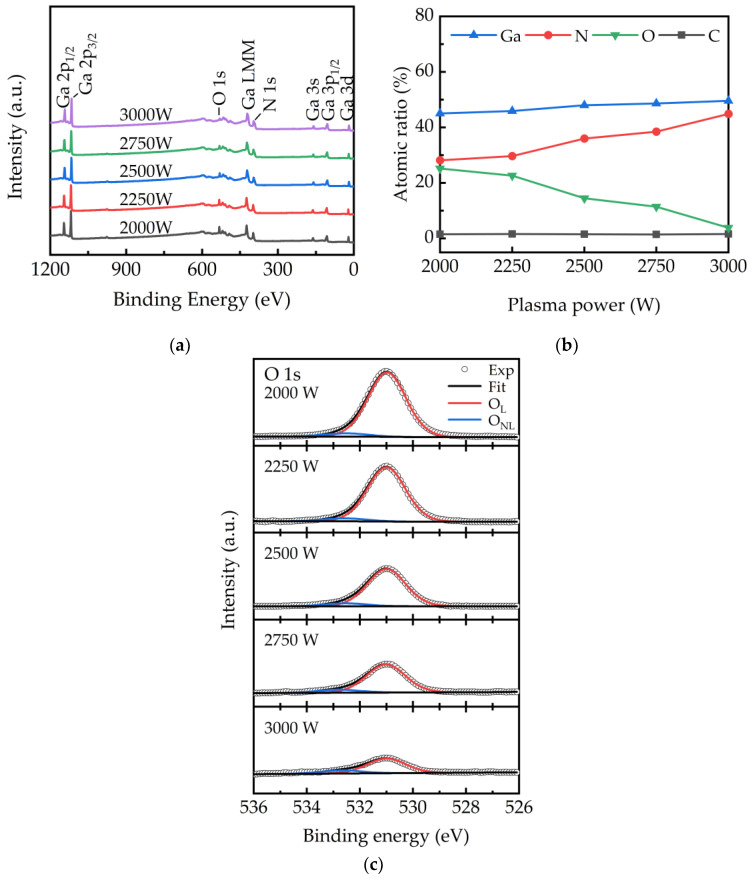
(**a**) The survey spectra, (**b**) the concentration, and (**c**) the O1s detail scan of GaN films obtained at different plasma power.

The surface chemical composition and bonding state of the PEALD-deposited GaN films was examined using XPS measurements. The XPS survey spectrum is shown in Figure 3a. Elements of Ga, O, N, and C were found in the GaN films. Quantitative analysis of Ga, O, N, and C varied with the plasma power are plotted as shown in Figure 3b. A minor C about 1 at% from the contamination of XPS measurement is observed. The Ga concentration is slightly affected by the variation of plasma powers. The nitrogen content increases from 28.21 to 44.86 at% while the oxygen content decreases from 25.21 to 3.89 at% when increasing the plasma power from 2000 to 3000 W. The observation of oxygen is attributed to the presence of residues or moisture in the chamber during deposition. The incorporation of oxygen along with the presence of amorphous Ga_2_O_3_ in PEALD-deposited GaN films cannot be avoided [36]. Moreover, it was observed that the oxygen content is the key to affect the atomic stoichiometry of the GaN film. In our study, the reaction cavity wall is bombarded by the plasma to generate oxygen species originated from residues gas in the chamber. The oxygen adsorbs on the sample surface and reacts with Ga like NH, NH_2_, and H radicals in the plasma. Since the Ga-O are more likely to form bonds than Ga-N, as shown in Figure 3b, the PEALD-deposited GaN films obtained at low plasma power are oxygen highly contained and more far away from the stoichiometry of GaN. At higher plasma power, the increase in H radicals leads to the dissociation of Ga-O bond and the formation of OH or H_2_O desorbed from the surface. Therefore, more adsorption sites on the sample surface are occupied by NH, NH_2_, and H radicals to grow GaN. As a result, by increasing the plasma power, the stoichiometry of GaN films is gradually satisfied. In other words, the H radicals increased by increasing the plasma power reduces the oxygen contamination in growing GaN film. Figure 3c shows the high-resolution XPS spectra of O1s. The O1s spectrum was resolved into two peaks at 531 and 532.8 eV, representing lattice oxygen and adsorbed O-H binding [37], respectively. The result confirms that as the plasma power increases from 2000 W to 3000 W, the significant decrease in the O1s peak is mainly due to the reduction of Ga-O bonds in the GaN lattice. Furthermore, the oxygen in GaN film is mainly in form of lattice oxygen with a few adsorbed O-H binding. The adsorbed O-H binding is attributed to the indefinite hydroxyl group of water that is inevitably adsorbed on the membrane under environmental conditions adventitious hydroxyls of water are inevitably adsorbed on films in ambient conditions [38], which is independent of plasma power.

**Figure 4:**
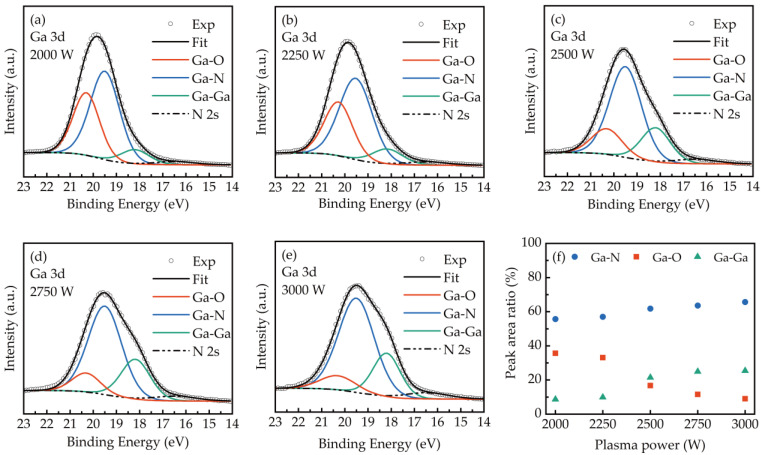
The high-resolution XPS spectra of Ga3d for GaN films obtained at (**a**) 2000 W, (**b**) 2250 W, (**c**) 2500 W, (**d**) 2750 W, and (**e**) 3000 W. (**f**) The bonding percentages of Ga-O, Ga-N, and Ga-Ga bonds in GaN films.

The variation of the Ga-O and Ga-N bonds in GaN films with the plasma power is further discussed by the high-resolution Ga3d spectra as shown in Figure 4a–e. The Ga3d was deconvoluted into four bonding states of Ga-O, Ga-N, Ga-Ga, and N 2s at 20.23, 19.5, 18.2, and 16.5 eV, respectively [39,40]. The bonding percentages of Ga-O, Ga-N, and Ga-Ga bonds were plotted in Figure 4f. It was observed that the bonding of Ga-N with a concentration from 55.7 to 65.6% is majority in the GaN films and increases with the increase of plasma power. The Ga-O bonds significantly reduced from 35.6 to 9% as the plasma power is higher than 2250 W. It also confirms that a higher plasma power leads to dissociate Ga-O bonds with H radicals and provides more adsorption sites for NH, NH_2_, and H radicals to grown GaN fims. In addition, the increase in Ga-Ga bond is attributed to the strong removal of Ga-O by H radical etching at a higher power plasma.

Since the oxygen also affects the growth of GaN films, the growth mechanism of GaN involving oxygen was established as follows:

Chemical reactions involving oxygen:

Ŝ-(N-Ga(CH_3_)_3-x_)_x_ + O → Ŝ-(N-Ga(OH)_3-x_)_x_ + CH_4 (g)_, (6)

Ŝ-(N-Ga(OH)_3-x_)_x_ + H → Ŝ-(N-GaH_3-x_)_x_ + H_2_O _(g)_, (7)

**Figure 5:**
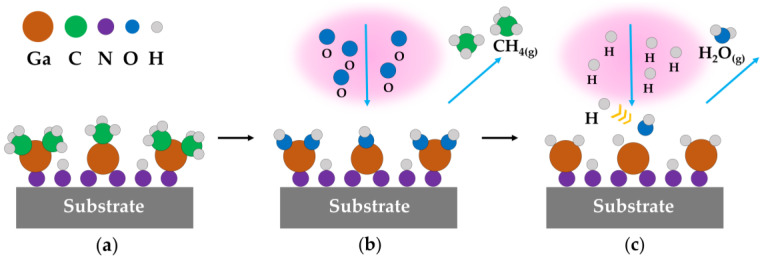
A schematic diagram of the mechanism described in Equations (6) and (7). (**a**) the surface of films after 1st half reaction, (**b**) oxygen radicals oxidize Ŝ-(N-Ga(CH_3_)_3-x_)_x_, (**c**) Ga-OH bond dissociation via hydrogen radicals.

As mentioned in Equation (3), the TMGa adsorbs on the surface and bonds with Ŝ-NH_2_ groups to produce Ŝ-(N-Ga(CH_3_)_3-x_)_x_ in the 1st half-reaction. Figure 5a illustrates the surface of the grown film after the 1st half-reaction. Then, with the presence of oxygen, Ŝ-(N-Ga(CH_3_)_3-x_)_x_ is oxidized by oxygen radicals to form Ŝ-(N-Ga(OH)_3-x_)_x_ as shown in Equation (6) and Figure 5b. Since the OES is placed above the reaction cavity, the emission of any O-species is not observed. Notably, the H radicals readily bond to the OH termini on the film surface [31]. Therefore, as shown in Equation (7) and Figure 5c, the H radicals selectively dissociates the Ga-OH bond on the surface of the sample to form H_2_O and desorb from the surface. This reaction mechanism confirms that with the increase of plasma power, the oxygen content in the GaN film decreases due to the increase of H radicals. Therefore, a relatively low oxygen level in the currently reported data, as low as 3.89 at%, were obtained in this study.

It is known that the oxygen not only comes from residues or moisture but also from plasma bombardment of the quartz chamber in the PEALD process [22,23]. The residues and moisture can be effectively reduced by cleaning and lowering background pressure. However, the oxygen generated by the plasma bombardment of the quartz chamber cannot be easily avoided. Table 1 lists the oxygen content reported in PEALD-prepared GaN films. Regardless of Ga precursor and N source type, the oxygen content ranges from 2.5 to 21.5 at%. It is found that most of the PEALD–GaN film was obtained by ICP plasma source in a quartz chamber. With the exception of a few studies, the oxygen content exceeded 9 at% [20,23,24,25,26]. It is noted that the oxygen content of PEALD–GaN film reported by P. Motamedi et al., was only 2.5%, however, oxygen contamination was not fully reported [27]. Instead of depositing their GaN in a quartz chamber, S. Kizir et al., used a hollow cathode plasma (HCP) source made of stainless steel, thus obtaining a low oxygen content of 3% [41]. In addition, the HCP-deposited GaN has a less Ga content and deviates from stoichiometric ratio of 1:1. M. Alevli et al., also reported the use of HCP source to obtain GaN films with low oxygen content [42,43,44]. However, it is noted that a higher oxygen content of 11 at% was observed when the plasma was generated using only N_2_ gas [45]. This also confirms our proposed mechanism of H Radical reducing oxygen content in the plasma. As the primary plasma source for depositing GaN thin films, ICP has excellent atomization, excitation, and ionization capabilities for commercial applications. The issue of eliminating oxygen contamination in the ICP process is absolutely critical. In our study, by increasing the power of the NH_3_+Ar plasma, the oxygen content was significantly decreased from 25.21 to 3.89 at%, which is relatively low compared to other reports. It is proposed that the capture of oxygen by H radicals through preferentially dissociating Ga-OH bonds during the 2nd half-reaction is the mechanism for reducing the oxygen content in the ICP process.

**Figure 6:**
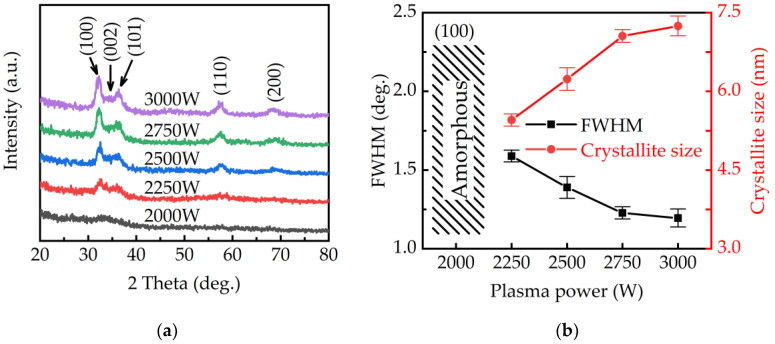
(**a**) The XRD spectra of GaN films and (**b**) the FWHM and crystalline size of GaN films obtained at different plasma powers.

Figure 6a shows the XRD patterns of GaN films obtained at different plasma powers. The diffraction peaks at 32.3°, 34.5°, 36.8°, 48°, 57.7°, and 67.8° correspond to the (100), (002), (101), (110), (200) planes of the GaN structure (JCPSD#50-7092). There is no diffraction peak of gallium oxide was observed. At a low plasma power of 2000 W, the diffraction peaks of GaN are weak and exhibits an amorphous structure. It is because abundant Ga-O bonds and poor plasma dissociation limit the growth of GaN at low plasma power. As the plasma power increases higher than 2250 W, clear diffraction peaks of GaN appear. To further understand the dependence of GaN crystallinity on plasma power, the crystalline size was calculated by extracting the full width at maximum height (FWHM) of the (100) orientation according to the Scherrer equation.

(8) D=Kλ/βcosθ,

where D is the crystallite size, K is the dimensionless shape factor (0.9), λ is the X-ray wavelength (0.154 nm), β is the FWHM, and θ is the Bragg diffraction angle. As shown in Figure 6b, increasing the plasma power from 2250 to 3000 W increases the crystallite size and crystallinity of the GaN films. The increase in the crystallinity of the film is due to the highly dissociated plasma and reduced Ga-O bonds. On the other hand, at high plasma power, the abundant H radicals in the highly dissociated plasma not only help to reduce the formation of Ga-O bonds on the surface, but also help to increase crystallinity through the etching effect.

**Figure 7:**
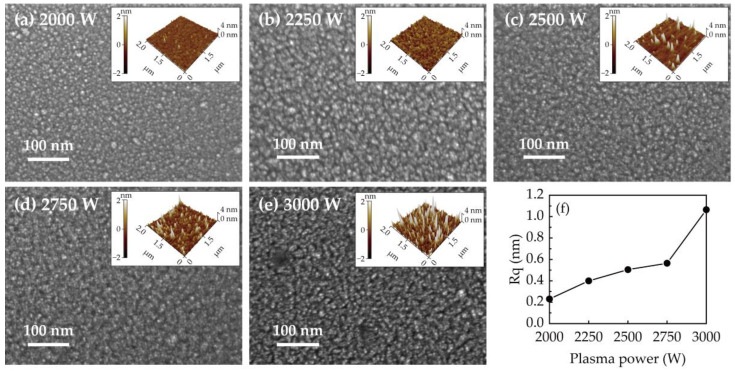
The FESEM image with a corresponding AFM image obtained at (**a**) 2000, (**b**) 2250, (**c**) 2500, (**d**) 2750, and (**e**) 3000 W. (**f**) The plot of RMS with the plasma powers.

In addition to crystallinity, surface morphology and roughness are also affected by the same effects. As shown in Figure 7a–e and the inserts, FESEM as well as the AFM are used to observe the morphology of PEALD–GaN obtained at different plasma powers. Figure 7f plots the root mean square roughness (RMS) with the plasma powers. The granular morphology was both observed by FESEM and AFM. At a low plasma power of 2000 W, the morphology exhibits smooth granularity with low roughness. By increasing the plasma power from 2250 to 3000 W, the boundaries around the particles become deeper as the RMS increases from 0.40 to 1.06 nm. The significant increase in RMS at 3000 W confirms that the selective etching of H radicals dominates the growth process at high plasma power.

**Figure 8:**
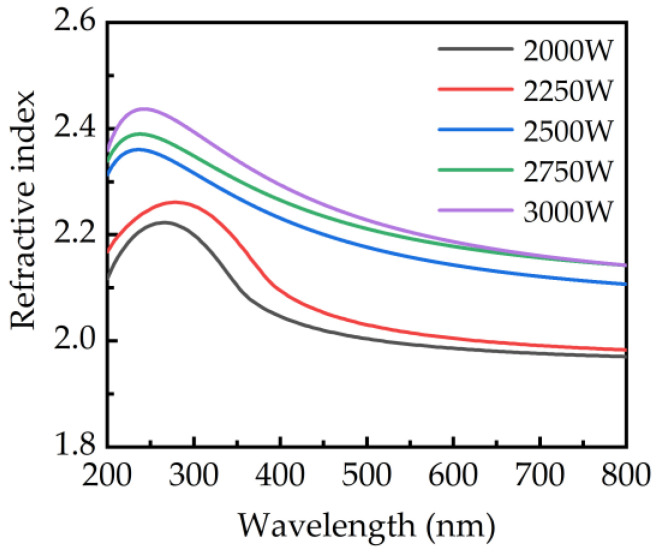
The refractive index of GaN films obtained at different plasma powers.

The optical properties of the PEALD–GaN obtained at different plasma power are measured. The refractive index is shown in Figure 8. The refractive index increases with increasing the plasma power. The refractive index obtained at 632.8 nm increases from 1.98 to 2.17 with increasing the plasma powers from 2000 to 3000 W. The refractive indices of GaN films obtained at plasma powers of 2000 and 2250 W is significantly lower than that obtained at plasma powers of 2500, 2750, and 3000 W. It is known that the refractive index is 1.92 for single-crystal Ga_2_O_3_ and 2.38 for single-crystal GaN at the wavelength of 632.8 nm [46,47]. As mentioned earlier, more Ga-O bonds were observed at low plasma power, which results in the refractive index of the PEALD–GaN being lower than that of GaN and close to that of Ga_2_O_3_. As the plasma power increases, H radicals reduce the Ga-O bonds and increase the film crystallinity. Thus, larger grain sizes and higher crystallinity lead to a higher refractive index of the PEALD–GaN [48], thereby increasing the refractive index close to that of GaN at high plasma power.

**Figure 9:**
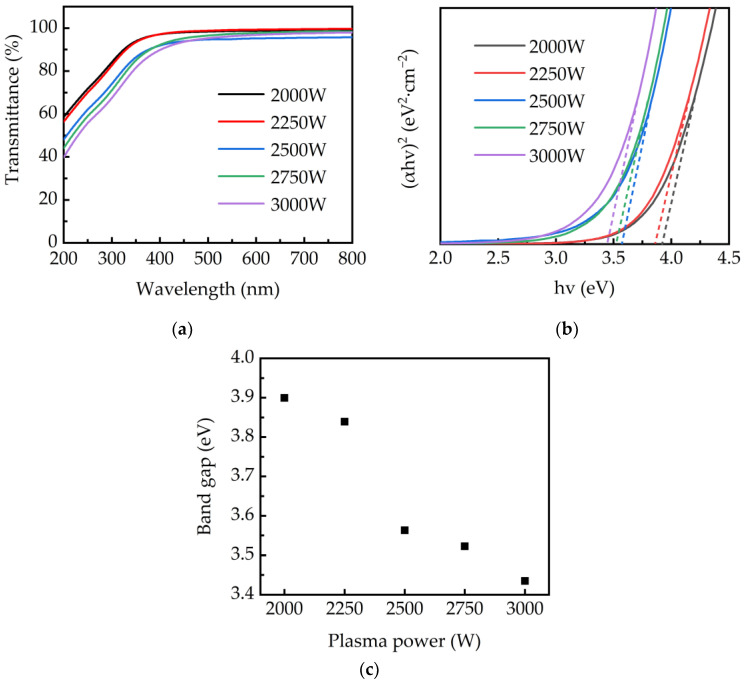
(**a**) Transmittance spectra of the PEALD-GaN deposited at various plasma powers. (**b**) The plot of (αhv)^2^ as a function of photon energy (hv). (**c**) The optical bandgap of the PEALD-GaN films.

Figure 9a shows the transmittance of PEALD–GaN films deposited on a sapphire substrate. It shows that the transmittance decreases with increasing plasma powers at wavelengths from 200 to 800 nm. This phenomenon is attributed to the decrease of the Ga-O bonds of the thin films with increasing plasma power. In addition, the absorption edge shows a redshift as the plasma power increases. The optical bandgap is, therefore, calculated and plotted in Figure 9b,c, respectively. The optical bandgap of the film is calculated using the Tauc plot method, described by the following Equation [49].

(9) $${\left(\alpha \mathrm{hv}\right)}^{n}=A\;\left({\mathrm{hv}-E}_{g}\right).$$

where α is the absorption coefficient, hv is the photon energy, A is the material correlation constant, n is 2 for direct bandgap material, and E_g_ is the band gap. The α is calculated by the Beer-Lambert law: α(λ) = ln(1/T(λ))/d, where T is the penetration rate and d is the thickness of the film, the thicknesses of the GaN films at plasma power of 2000, 2250, 2500, 2750, and 3000 W are 35.9, 36.9, 41.1, 41.7, and 41.8 nm, respectively. The band gap of the PEALD–GaN films was obtained by extrapolating the linear region of (αhν)^2^ to hν to the horizontal axis, as shown in Figure 9b. It was observed that the band gap decreases from 3.89 eV to 3.43 eV with increasing plasma power. A higher bandgap ranging from 3.89 to 3.82 eV was obtained at the plasma power at 2000 to 2250 W. At plasma power at 2750 and 3000 W, the bandgap significantly decreases to 3.52 and 3.43 eV, respectively. Furthermore, the significant decrease in the bandgap has the same reason with the significant increase in the refractive index as increasing the plasma power.

**Figure 10:**
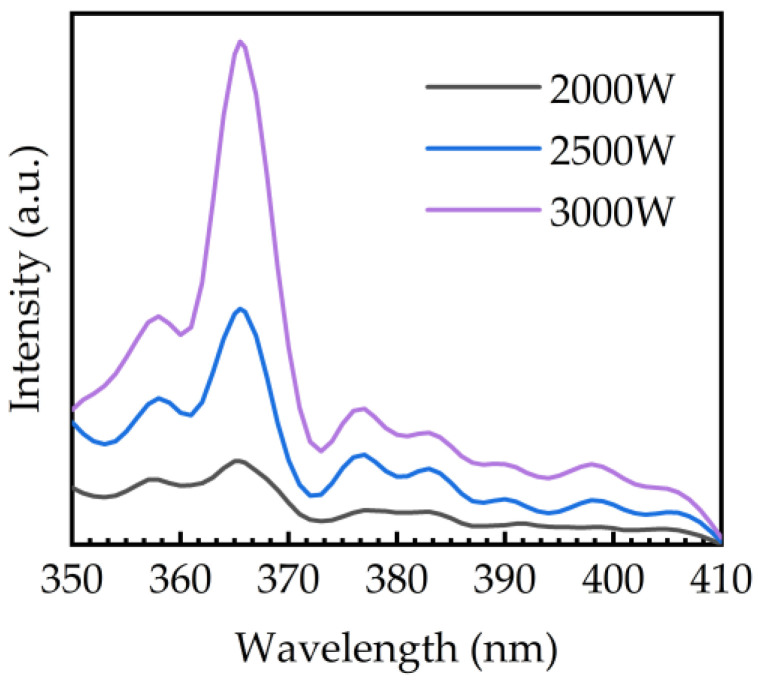
The PL spectra of PEALD-GaN films obtained at plasma power of 2000, 2500, and 3000 W.

The photoluminescence spectra of the PEALD–GaN film deposited on Si wafer was measured as shown in Figure 10. In our study, the PEALD–GaN films were mainly polycrystalline, so other luminescence peaks at 358, 377, 383, and 398 nm also appeared along with 365 nm. The most prominent 365 nm peak, close to the band edge of GaN, was originated form the exciton recombination [50]. The free exciton peak at 358 nm is observed [51]. In addition, other luminescence peaks attributed to defects luminescence appeared at 377, 383, and 398 nm. Among them, the peak at 383 nm is considered to result from the recombination of an exciton bound to point defect [52]. The peaks at 377 nm and 398 nm are associated with neutral shallow donor bound exciton [53]. Furthermore, an increase of luminescence intensity was observed due to the enhanced crystallinity and reduced oxygen content of GaN thin films by H radicals obtained at high plasma powers.

## 3. Materials and Methods

**Figure 11:**
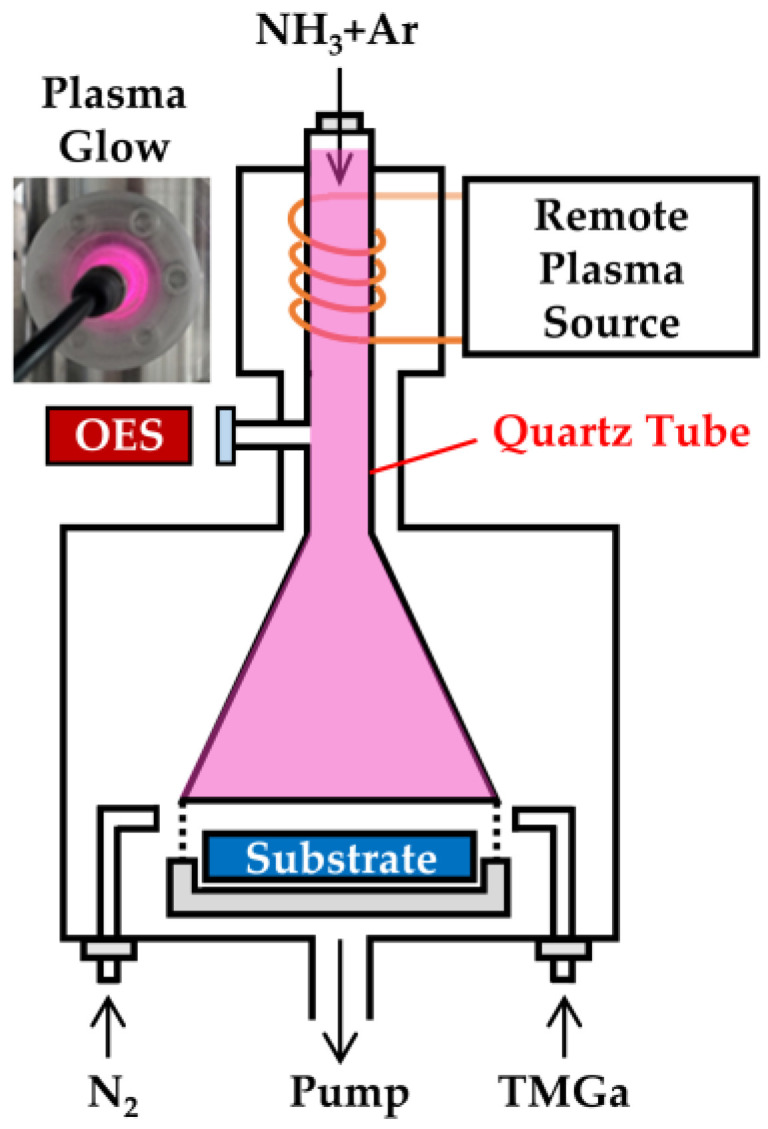
The schematic plot of the deposition chamber and OES detector location.

The GaN thin film was deposited on p-type (100) Si wafer and sapphire substrate by a PEALD system (Picosun R-200, Espoo, Finland). Before deposition, the silicon wafers were cleaned using standard Radio Corporation of American (RCA) procedures and then dipped in a 2% HF solution for 1 min to remove the native oxide from surface. Finally, the Si wafers were rinsed in deionized water and dried with N_2_. The sapphire substrates were cleaned for 15 min with deionized water, ethanol, isopropyl alcohol, and deionized water in sequence, and then dry with N_2_. For the GaN deposition, trimethylgallium (TMGa, Ga (CH_3_)_3_, 99.9999%, Nanjing Ai Mou Yuan Scientific Equipment, Nanjing, China) and NH_3_ were used as the precursor of Ga and N, respectively. TMGa was stored in stainless steel bottles at 0 °C and carried by 120 sccm N_2_ gas. As shown in Figure 11, the plasma in a microwave cavity was generated by RF inductively coupled plasma power (ICP, Litmas RPS, Advanced Energy, Denver, CO, USA) in a mixture of 160 sccm Ar (99.999%) and 30 sccm NH_3_ (99.999%) gases. The RF power was controlled in the range of 2000–3000 W. The GaN growth cycle in the PEALD system was (i) TMGa with a pulse time of 0.1 s, (ii) N_2_ purge for 4 s, (iii) NH_3_/Ar plasma treatment for 13 s, and (iv) N_2_ purge for 6 s. The total cycle numbles are fixed at 1000. During deposition, the substrate temperature was controlled and fixed at 350 °C. The detailed growth parameters are listed in Table 2.

During the deposition, as schematically shown in Figure 11, the optical emission spectrometer (OES, SD2048DL, Verity, Carrollton, TX, USA) was used to diagnose the plasma. The spectroscopic ellipsometer (SE, SENTECH SE 800 DUV, Berlin, Germany) was used to examine the thickness and refractive index of the GaN films deposited on silicon wafers. The refractive index was obtained using a Tauc–Lorentz model. The chemical compositions and bonding states were characterized with X-ray photoelectron spectroscopy (XPS, ESCALAB 250Xi, Thermo Fisher, Waltham, MA, USA) with a monochromatized Al Kα X ray source (spot size 400 μm^2^). For the instrumental calibration, the binding energies of the XPS spectra were calibrated by taking the peak positions of Au (83.942 eV), Cu (932.626 eV), and Ag (368.211 eV) as a standard reference before testing. High-resolution XPS data was corrected for charging by shifting peaks with respect to the adventitious C 1s peak located at 284.84 V. The microstructure of the GaN films were examined with conventional θ–2θ X-ray diffraction (XRD, Rigaku TTRAXIII, Ibaraki, Japan) using a Cu Kα emission line. The surface morphology of GaN films were examined via atomic force microscopy (AFM, Bruker) and field emission scanning electron microscopy (FESEM, JSM-7800F, JEOL, Tokyo, Japan). The optical transmittance of the films deposited on the sapphire substrates was obtained with a UV-vis spectrophotometer (Lambda850, PerkinElmer, Waltham, MA, USA) in wavelength ranging from 200 to 800 nm. The steady-state photoluminescence (PL) spectra were measured with a fluorescence spectrometer (Edinburgh FLS 980, Livingston, UK) using a xenon lamp with an excitation wavelength of 325 nm.

## 4. Conclusions

GaN thin films was deposited by PEALD using TMGa metal source and NH_3_/Ar plasma at powers ranging from 1500 to 3000 W. At a low power of 1500 W, the low efficiency of the plasma dissociation was too low to grow GaN films. At powers higher than 1500 W, the emission spectra show that the emission intensity of H, NH, and NH_2_ radicals increase with plasma powers. Among them, the H emission increases linearly and has the most pronounced increase compared to NH and NH_2_ emissions. At high powers, the dissociation of NH_2_ becomes saturated, and the dominate H radical has an etching effect, and thus, the growth rate of GaN film is saturated. The oxygen incorporation into the grown GaN film to form Ga-O bonds is unavoidable in the PEALD process using an ICP plasma source. In the presence of oxygen, the 2nd half-chemical reaction takes a route to form Ŝ-(N-Ga(OH)_3-x_)_x_ instead of Ŝ-(N-GaH_3-x_)_x_. However, the H radicals dissociated from the NH_3_-containing plasma can capture the oxygen on the surface of the grown film and eliminate Ga-O bonds to form Ŝ-(N-GaH_3-x_)_x_. Then, the NH and NH_2_ radicals replace the H in Ŝ-(N-GaH_3-x_)_x_ to form Ŝ-(N-Ga(NH_2_)_3-x_)_x_ leading to the growth of GaN monolayer. Therefore, the H radicals obtained at high plasma power leads to the low ratio of Ga-O observed in the XPS results. As a result, by increasing the plasma power, GaN films with better crystallinity are close to stoichiometric. This study proposes and establishes the mechanism that increasing the free radical of H in the NH_3_+Ar plasma can reduce the oxygen contamination of PEALD–GaN films and also solves the problem of high oxygen contamination in ICP-PEALD. Furthermore, the decrease of the Ga-O bonds of the thin films with increasing plasma power leads to the increase of the refractive index and the reduction of the bandgap. The polycrystalline GaN with low oxygen contents obtained at high plasma power shows high luminescence intensity.

## Figures and Tables

**Table 1:** The oxygen content reported in PEALD-prepared GaN films.

Precursor	Gas	Power	Pressure (Torr)	O Content (at.%)	Ref.
TEGa	N_2_/H_2_	HCP	0.15	3	[41]
TMGa	N_2_/H_2_	HCP	-	3.18	[42]
TEGa	N_2_/H_2_	HCP	-	3.25	[43]
TEGa	N_2_/H_2_	HCP	-	1.71	[44]
TMGa	N_2_	HCP	-	11	[45]
TEGa	N_2_/H_2_/Ar	ICP	0.16	11.61	[23]
TEGa	N_2_/H_2_/Ar	ICP	0.4	9	[24]
TMGa	NH_3_/Ar	ICP	0.15	21.46	[25]
TEGa	N_2_/H_2_/Ar	ICP	0.15	20	[20]
TEGa	NH_3_/H_2_/Ar	ICP	0.4	13	[26]
TEGa	N_2_/H_2_	ICP	-	2.5	[27]
TMGa	NH_3_/Ar	ICP	0.75	3.89	This work

Triethylgallium [TEGa, Ga(C_2_H_5_)_3_].

**Table 2:** Detailed deposition parameters of the GaN films.

Parameters	Values
Bubbler temperature (°C)	0
Substrate temperature (°C)	350
TMGa pulse time (s)	0.1
N_2_ carry rate for TMGa enters (sccm)	120
N_2_ purge time after TMGa (s)	4
NH_3_/Ar plasma treatment (s)	13
N_2_ purge time after NH_3_/Ar plasma (s)	6
Ar flow rate (sccm)	160
NH_3_ flow rate (sccm)	30
NH_3_/Ar plasma power (W)	1500–3000

## Data Availability

Not applicable.
